# Beyond Geography: Climatic Gradients Shape Reeves's Muntjac Population Structure in Taiwan

**DOI:** 10.1002/ece3.73283

**Published:** 2026-03-18

**Authors:** Yi‐Lun Peng, Hsuan‐Wien Chen, Chun‐Yi Hsiao, Shih‐Wei Chang, Hurng‐Yi Wang

**Affiliations:** ^1^ Institute of Ecology and Evolutionary Biology National Taiwan University Taipei Taiwan; ^2^ Department of Biological Resources National Chiayi University Chiayi Taiwan; ^3^ Technology Commons, College of Life Science National Taiwan University Taipei Taiwan; ^4^ Taiwan Biodiversity Research Institute Jiji Nantou Taiwan; ^5^ Graduate Institute of Clinical Medicine, College of Medicine National Taiwan University Taipei Taiwan; ^6^ Graduate Institute of Medical Genomics and Proteomics, College of Medicine National Taiwan University Taipei Taiwan; ^7^ Department of Entomology National Taiwan University Taipei Taiwan

## Abstract

Understanding how geographic and climatic gradients shape genetic architecture is a central goal of evolutionary ecology. In Taiwan, mammals show varied divergence: low‐mobility species such as mole‐shrews and Formosan wood mice exhibit strong north–south splits, and surprisingly, similar patterns occur in mobile taxa like Formosan serow and sambar deer. In contrast, other mobile species, including flying squirrels and Reeves's muntjac, show weak or no population structure in prior studies. This recurring north–south divergence across ecologically diverse taxa suggests that shared environmental gradients, beyond historical isolation, drive parallel population structures. If so, species occupying similar habitats may exhibit comparable genetic breaks regardless of life‐history traits. Prior mitochondrial studies likely missed fine‐scale structure in muntjac; high‐resolution SNP data now offer improved resolution. Here, we analyzed genome‐wide SNPs from 71 Taiwanese Reeves's muntjac and comparative Chinese samples. We detected deep divergence from Chinese muntjac (~0.24 MYA), and further north–south subdivision within Taiwan (~0.06 MYA). Demographic modeling revealed a complex history involving glacial isolation and asymmetric gene flow, mainly from north to south. Within Taiwan, genetic differentiation was shaped by both geography and climate, especially temperature annual range (Bio7), with niche models showing environmental separation. Selection scans identified PLA2‐associated genes, potentially linked to thermal adaptation. This is the first study to demonstrate that both geographic and environmental heterogeneity jointly contribute to mammalian divergence in Taiwan. The repeated north–south split across ecologically diverse species highlights shared climatic and topographic factors driving parallel population structure in Taiwan's montane ecosystems.

## Graphical abstract

North–south population split in Reeves's muntjac revealed by genome‐wide SNPs. Parallel divergence patterns across mammals in Taiwan suggest shared environmental drivers. Demographic modeling supports historical isolation and asymmetric gene flow. Genetic differentiation shaped by geographic and climatic gradients, especially temperature range. Selection scans identify PLA2‐related genes linked to thermal adaptation.

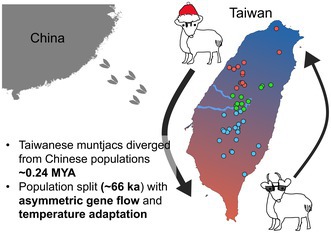

## 1 Introduction

Understanding genetic diversity and differentiation within species is crucial for biodiversity conservation, as it underpins adaptability and ecosystem resilience. Analyzing genetic patterns helps identify hotspots of variation, informs targeted conservation strategies, and supports habitat restoration. These insights are particularly important for developing climate‐resilient conservation plans (Hughes et al. 2008; Allendorf et al. 2010; Hoban et al. 2021; Shaw et al. 2025). Genetic differentiation in mammals is shaped by historical processes (e.g., glacial cycles), environmental variation (e.g., habitat and climate), and species‐specific traits such as dispersal and demography (Clobert 2012; Wang and Bradburd 2014; Theodoridis et al. 2020; Afonso Silva et al. 2025). In this context, Taiwan provides a valuable natural laboratory, where diverse ecological settings and complex topography have given rise to a wide range of phylogeographic patterns across mammalian species.

Genetic studies of Taiwanese mammals have revealed divergent patterns of population structure that appear linked to dispersal ability. Species with limited dispersal, such as the mole‐shrew ( *Anourosorex yamashinai* ) and Formosan wood mouse ( *Apodemus semotus* ), show distinct northern and southern lineages (Hsu et al. 2001; Yuan et al. 2006), with the boundary broadly corresponding to the Wu (Dadu) and Zhoushui Rivers. Interestingly, some large mammals with no apparent dispersal restriction like Formosan serow ( *Capricornis swinhoei* ) and sambar deer ( *Rusa unicolor swinhoii* ) also exhibit similar north–south divergence (Horng et al. 2003; Li et al. 2023). In contrast, species with strong dispersal ability, including the white‐bellied rat ( *Niviventer culturatus* ), Formosan Reeves's muntjac ( *Muntiacus reevesi micrurus* ), and flying squirrels (*Petaurista lena* and *P. grandis* ), exhibit weak or no population structure (Hsu et al. 2000; Chan 2004; Oshida et al. 2011; Tan 2017).

Distinct mitochondrial DNA (mtDNA) lineages observed in various taxa are proposed to have originated from Pleistocene glacial refugia (Oshida et al. 2006; Yuan et al. 2006; Y.‐T. Wang 2012). For example, the divergence between major phylogroups of the mole‐shrew was estimated at 0.63–0.71 MYA (Yuan et al. 2006), while the two Formosan sambar deer clades coalesced more recently in the late Pleistocene, approximately 0.21–0.63 MYA (Li et al. 2023). Although glacial refugia might have promoted genetic divergence, subsequent post‐glacial dispersal likely blurred their geographic patterns. Species like the sambar deer, adapted to steep terrain, may traverse steep landscapes effectively and retain multiple mitochondrial lineages within local populations. In addition, these estimates rely on standard mutation rates that do not account for species‐specific demographic traits, such as generation time, which can significantly influence the detectability and calibration of genetic signatures of historical events. Thus, the recurrence of broadly similar north–south patterns across taxa with contrasting dispersal capacities suggests that historical isolation alone cannot fully explain the observed differentiation and warrants further investigation. At the same time, most existing inferences rely on single‐locus mtDNA, making it difficult to determine whether “weak structure” reflects true biological homogeneity or limited marker resolution. Together, these limitations motivate re‐evaluating “weak structure” taxa with higher‐resolution genomic data to disentangle signals of historical isolation from those driven by contemporary ecological processes.

In addition to historical processes, contemporary geographic and environmental factors may also contribute to phylogeographic structure. Taiwan spans a pronounced north–south climatic transition from subtropical to tropical conditions, coupled with steep elevational relief that generates strong gradients in temperature variability and precipitation seasonality under the East Asian monsoon system. These environmental contrasts shape forest composition, phenology, and resource predictability across elevations, creating repeated opportunities for both geographic isolation and environment‐dependent connectivity. Even in the absence of absolute dispersal barriers, such climatic discontinuities can reduce effective gene flow through isolation by environment (IBE), via habitat preference, reduced establishment of migrants, or local adaptation, leading to genetic breaks that align more closely with climate than with geographic distance (Sexton et al. 2014; Wang and Bradburd 2014). The Reeves's muntjac represents a “medium‐dispersal” model; for such species, we hypothesize that sharp climatic transitions act as selective filters or physiological barriers that restrict gene flow through IBE, even where absolute physical barriers are absent. If this is the case, species with similar distributions should exhibit comparable north–south splits, regardless of their historical background. Comparing the muntjac's genomic patterns with those of other mammals showing similar north–south breaks can further test whether environmental heterogeneity consistently promotes divergence across Taiwanese taxa. Prior studies may have overlooked fine‐scale patterns in species such as Reeves's muntjac, due to reliance on mtDNA, which reflects only maternal lineages and may miss subtle population differentiation (Hurst and Jiggins 2005). By using genome‐wide SNP data, we can resolve phylogeographic patterns and identify the ecological and genetic factors shaping intraspecific divergence across Taiwan's montane ecosystems.

To further investigate the drivers of population divergence in Taiwan's montane fauna, we analyzed the genomic variation of the Taiwanese Reeves's muntjac using double digest Restriction‐site Associated DNA sequencing (ddRAD‐seq). Our results revealed a clear genetic division between northern and southern populations, with the boundary broadly corresponding to the Wu (Dadu) and Zhoushui Rivers. This north–south split is associated with environmental variables, particularly temperature annual range, implying that local climatic conditions, in addition to geographic barriers, contribute to population structure. We also noted that this genetic break mirrors those observed in other mammals, such as the Formosan serow and sambar deer, indicating that shared climatic and geographic gradients may drive parallel divergence across species in Taiwan's mountainous landscapes.

## 2 Materials and Methods

### 2.1 Sample Collection, DNA Extraction and Sequencing

**FIGURE 1:**
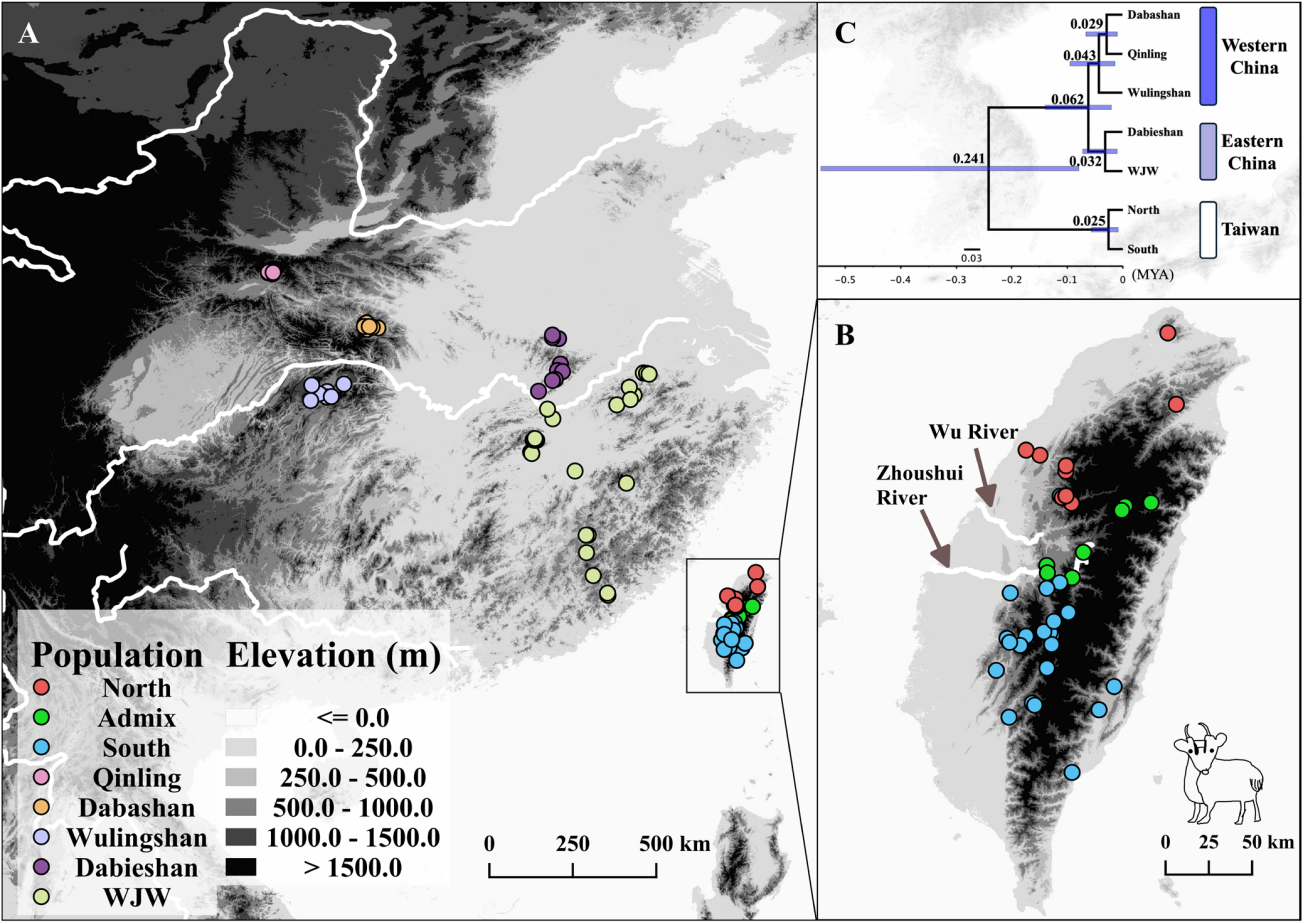
Sampling locations and phylogenetic relationships of Reeves's muntjac. (A) Sampling localities of Reeves's muntjac from Taiwan and China. Chinese populations are categorized into eastern (WJW: Wuyishan, Wannan, and Jiulingshan; Dabieshan) and western (Qinling, Dabashan, and Wulingshan) clusters, following Chen et al. (2024). Taiwanese populations are divided into three genetically distinct groups: Northern (pink), admixed (green), and southern (blue). (B) Sampling localities of Taiwanese Reeves's muntjac ( *Muntiacus reevesi micrurus* ) show three genetic clusters: Northern (pink), admixed (green), and southern (blue). (C) Species tree inferred using Bayesian coalescent analysis with SNAPPER. Median divergence times (with 95% confidence intervals) are shown above nodes. All posterior probabilities exceed 0.9.

A total of 71 tissue samples of Taiwanese Reeves's muntjac ( *Muntiacus reevesi micrurus* ) were provided by the Taiwan Biodiversity Research Institute (Letter from the Endemic Species Research Institute, COA, Executive Yuan, dated October 18, 2022, Ref. No. Nong‐Te‐Dong‐Zi 1111002124) (Figure 1, Table S1). These samples were broadly representative of the species' habitats across Taiwan, spanning major geographic regions to ensure comprehensive coverage for population genomic analysis. Genomic DNA was extracted from muscle tissue using the FavorPrep DNA Extraction Mini Kit (Favorgen Biotech, Taiwan). The ddRAD libraries were constructed by the Technology Commons (National Taiwan University) following their standard protocol using SbfI and MseI restriction enzymes, and sequenced on an Illumina NovaSeq 6000 (150 bp paired‐end; Genomics BioSci & Tech, Taiwan). The mitochondrial cytochrome *b* gene was amplified using species‐specific primers (F: 5′‐ACCACGACTAATGATATGAAAAACC‐3′; R: 5′‐TGTCCTCCTTTTCTGGTTTACAA‐3′) and sequenced following PCR using the KAPA Taq Kit under standard cycling conditions.

### 2.2 Data Processing

Raw reads were processed using Stacks v2.62 (Rochette et al. 2019) with quality filtering via process_radtags (‐‐score‐limit 20). Filtered reads were aligned to the *M. reevesi* reference genome (GCA_008787405.2) using BWA‐MEM, and loci were assembled with gstacks, retaining paired‐end reads with mapping quality ≥ 20. SNPs were called with populations, retaining loci present in ≥ 80% of individuals and exported in VCF format (complete SNP dataset).

Whole‐genome sequences of Chinese *M. r. reevesi* were downloaded (Chen et al. 2024) and mapped to the reference genome (GCA_008787405.2) using GPU‐accelerated FQ2BAM workflow in NVIDIA Parabricks (NVIDIA 2019). Variants were called with bcftools (‐q 20 and ‐Q 20) (Danecek et al. 2021), and sites with depth ≥ 5 and < mean + two standard errors were retained. Regions homologous to the ddRADseq loci of Taiwanese muntjac, including both variant and invariant sites, were extracted to ensure consistency in downstream analyses.

Mitochondrial genomes were assembled from whole‐genome data using NOVOPlasty v4.3.5 (Dierckxsens et al. 2017), aligned with MARS (Ayad and Pissis 2017), and the cytochrome b gene was extracted for phylogenetic comparison with Taiwanese muntjac.

### 2.3 Population Structure Analysis

To minimize linkage bias, SNPs with minor allele count < 5 were excluded using VCFtools v0.1.17 (Danecek et al. 2011). Linkage disequilibrium (LD) pruning was guided by a GAM‐fitted LD decay curve (Wickham 2016), and SNPs with *r* ^2^ > 0.2 within 25 kb windows were removed using PLINK v1.90 (Purcell et al. 2007), yielding a pruned dataset for population structure analyses. Population structure was inferred using the LD‐pruned SNP set. ADMIXTURE v1.3.0 (Alexander et al. 2009) was run across *K* = 1–10, with cross‐validation to identify the optimal *K*. Principal component analysis (PCA) was performed using adegenet v2.1.10 (Jombart 2008), which depends on ade4 v1.7‐22 (Dray and Dufour 2007), and visualization of the results using ggplot2. Pairwise *F*_ST_ (Weir and Cockerham 1984) and nucleotide diversity (*π*) were calculated in VCFtools.

### 2.4 Phylogenetic Reconstruction and Divergence Time Estimation

Phylogenetic relationships were reconstructed using both nuclear and mitochondrial datasets. A maximum likelihood tree based on genome‐wide SNPs was inferred using IQ‐TREE2 v2.2.2.6 (Minh et al. 2020), with the best‐fit model selected by ModelFinder (Kalyaanamoorthy et al. 2017) and node support assessed via 10,000 ultrafast bootstrap replicates. Mitochondrial phylogeny was inferred from 943 bp of cytochrome b sequences from Taiwanese and Chinese muntjacs, supplemented with public data. Sequences were aligned using MUSCLE (Edgar 2004) in MEGA X (Kumar et al. 2018), and a neighbor‐joining tree was constructed under the Kimura 2‐parameter model.

Divergence times were estimated using Bayesian inference of species trees implemented in SNAPPER (Stoltz et al. 2021). To improve computational efficiency, 2000 SNPs and 10 individuals per population were subsampled following the recommendations of Chen et al. (2024). Analyses were run in BEAST2 (Bouckaert et al. 2019) for 1000,000 MCMC iterations, discarding the first 100,000 as burn‐in. Divergence times were calibrated using the median estimate for western Chinese populations (0.065 MYA; 95% CI: 0.003–0.154 MYA) and reported as medians with 95% highest posterior density (HPD) intervals.

### 2.5 Demographic Inference of Taiwanese Reeves's Muntjac

To infer the demographic history of Taiwanese muntjacs, site frequency spectrum (SFS) was generated from the complete dataset using easySFS (https://github.com/isaacovercast/easySFS), assuming a mutation rate of 2.38 × 10^−9^/site/generation (Yin et al. 2021) and a generation time of 2.5 years (Pacifici et al. 2013). Stairway Plot (Liu and Fu 2020) was used to infer independent demographic histories for northern and southern populations. To explore more complex demographic scenarios involving population size changes and gene flow, 12 models were evaluated using fastsimcoal2 v2.7.0.9 (Excoffier et al. 2021). The 12 demographic models evaluated in this study are initially categorized into two broad groups based on whether population size remained constant or changed following divergence. Within these two groups, the models are further distinguished by varying scenarios of post‐divergence gene flow, including no gene flow, early or recent gene flow, constant gene flow, gene flow rate changes, or interrupted gene flow (Figure S1). Divergence times were constrained between 20,000 and 100,000 years, based on SNAPPER estimates. Each model was run 100 times, with 40 expectation–conditional maximization (ECM) cycles and 100,000 simulations per cycle. The best‐fitting model was selected based on maximum likelihood using fsc‐selectbestrun.sh, and further evaluated using AIC via calculateAIC.sh (https://github.com/speciationgenomics/scripts).

Given the limited resolution of SFS for recent events, we used GONE (Santiago et al. 2020), a linkage disequilibrium‐based method, to infer changes in effective population size over the past 100 generations, assuming a recombination rate of 1 cM/Mb consistent with mammalian averages (Dumont and Payseur 2008).

### 2.6 Landscape and Climatic Correlates of Genetic Differentiation

To evaluate the drivers of genetic differentiation in Taiwanese Reeves's muntjacs, we examined the effects of geographic distance (straight‐line distance), landscape resistance (least‐cost path distance), and climatic variation (environmental dissimilarity) using 46 individuals with precise coordinates (one per locality) (Table S1). Genetic distances (Euclidean) were calculated using the gl.dist.ind in *dartR* v2.9.7 (Gruber et al. 2018). Geographic distances were computed as straight‐line distances using distm from *geosphere* v1.5‐18 (https://github.com/rspatial/geosphere). Least‐cost path distances were generated from a resistance surface combining slope and land‐use data. Slope layers were generated in QGIS (QGIS Development Team 2009) with resistance values increasing with gradient (0°–10° = 1, 20°–40° = 1.5, 50°–60° = 4, 70°–80° = 6, > 80° = 10). Land‐use categories (Chen et al. 2019) were similarly assigned values reflecting presumed resistance to muntjac movement: Forest = 1, Grassland = 2, Bare land = 2, Agricultural land = 3, Inland water = 5, Built‐up land = 10 (Wang 1989; McCullough et al. 2000). Final distances were computed with gl.costdistances in *dartR*. Climatic data were obtained from WorldClim v2, which includes 19 bioclimatic variables representing current climate conditions (Fick and Hijmans 2017). These widely adopted variables facilitate comparison with prior studies and can be projected onto paleoclimate reconstructions to evaluate historical versus contemporary suitability. Pearson's correlation coefficients among the 19 bioclimatic variables were calculated using raster.cor.matrix in ENMTools v1.1.2 (Warren et al. 2021). For variable pairs with |*r*| > 0.7, only the variable more strongly correlated with genetic distance was retained (Merow et al. 2013). Selected variables were used to calculate environmental dissimilarity with vegdist in *vegan* (Oksanen et al. 2024). Associations between genetic distance and geographic, resistance, and climatic distances were tested using Mantel and partial Mantel tests, with sampling year as a covariate to control for temporal effects.

### 2.7 Environmental Drivers of Genetic Structure and Niche Differentiation

Climatic variables significantly associated with genetic distance were used to quantify niche differentiation between northern and southern populations. PCA was conducted using *dudi.pca* in *ade4*, and population niches were modeled with ecospat.grid.clim.dyn in ecospat v4.0.0 (Di Cola et al. 2017). Niche equivalency and similarity (ecospat.niche.equivalency.test, ecospat.niche.similarity.test) were tested with 999 permutations to assess whether the two populations occupy distinct climatic spaces. To evaluate environmental influences on genetic structure, Generalized Dissimilarity Modeling (GDM; gdm v1.5.0‐9.1) (Mokany et al. 2022) was used to estimate the contributions of geographic distance, climate, and landscape resistance to genetic differentiation.

For distribution modeling, we used Maxent v3.4.4 (Phillips and Dudík 2008) with GPS data from sampled individuals and GBIF records (GBIF.org 2024). After removing duplicates, oceanic/erroneous points, and records with > 500 m coordinate uncertainty, 371 locality points were retained. We used QGIS v3.28.2 (QGIS Development Team 2009) to set spatial boundaries between 21.708°–26.5° N and 118°–123° E. We excluded highly correlated predictors (|*r*| > 0.7) and retained three Bioclim variables (Bio2, Bio7, and Bio15) for subsequent modeling. We also used Bioclim layers from the Last Glacial Maximum (LGM, ~22,000 years ago) (Hijmans et al. 2005) to reconstruct the species' potential distribution during glacial‐period.

### 2.8 Identification of Putative Loci Under Selection

We used two complementary approaches to identify loci putatively under selection. PCadapt v4.3.5 (Privé et al. 2020) identified SNPs strongly associated with principal components of genetic variation (FDR < 0.01), and BayeScan v2.1 (Foll and Gaggiotti 2008) detected outliers based on allele frequency differences under a Bayesian framework, using 20 pilot runs × 5000 iterations, burn‐in = 50,000, and prior odds = 100. Genes within ±150 kb of each candidate SNP were selected as candidates. From the reference genome (GCA_008787405.2), 23,112 protein‐coding genes were curated using AGAT v1.4.1 (Dainat et al. 2024), as the background for enrichment analysis. Functional annotation was performed using EGGNOG‐mapper v2.1.12 (Cantalapiedra et al. 2021). Gene Ontology (GO) enrichment was conducted using clusterProfiler v4.10.1 (Wu et al. 2021), using an adjusted *q* < 0.05. Candidate gene functions were inferred by identifying *Mus musculus* orthologs via Ensembl (Harrison et al. 2024), and phenotype associations explored using modPhEA (Weng and Liao 2017).

## 3 Results

### 3.1 Population Structure

We sequenced 71 Taiwanese Reeves's muntjac ( *Muntiacus reevesi micrurus* ) individuals from across Taiwan (Figure 1, Table S1), generating approximately 61.9 Gb of sequencing data. The average number of bases per individual was 756 Mb, with a standard deviation of 123 Mb (Table S2). Using bwa mem and gstacks, we obtained 524,360 loci, with an average sequencing depth of 60.3× per sample (standard deviation: 9.6×, with maximum and minimum depths of 89.5× and 35.3×, respectively) (Table S3). After filtering with populations, 37,878 loci were retained, containing a total of 94,034 SNPs (complete dataset) (Table S4). To further investigate the evolutionary history of Taiwanese Reeves's muntjac, 60 whole‐genome sequences of *M. reevesi reevesi* from China were downloaded, and genomic regions overlapping with our ddRAD‐seq loci were retained and combined with the Taiwanese ddRAD‐seq data for downstream analyses (see Section 2).

Compared to other deer species, Reeves's muntjac exhibits lower nucleotide diversity (*π*) (Table S5) (Hu et al. 2019; de Jong et al. 2021; Combe et al. 2022). In addition, the nucleotide diversity of Taiwanese Reeves's muntjac (0.26%) is approximately 40% less than that of Reeves's muntjac from China (0.42%–0.49%) (*p* < 10^−3^; permutation test). The nucleotide diversity of Reeves's muntjac from China, estimated from whole‐genome data, closely matches that of the subset overlapping with our ddRAD‐seq loci (Table S5), suggesting that these loci are representative of the genome‐wide diversity.

**FIGURE 2:**
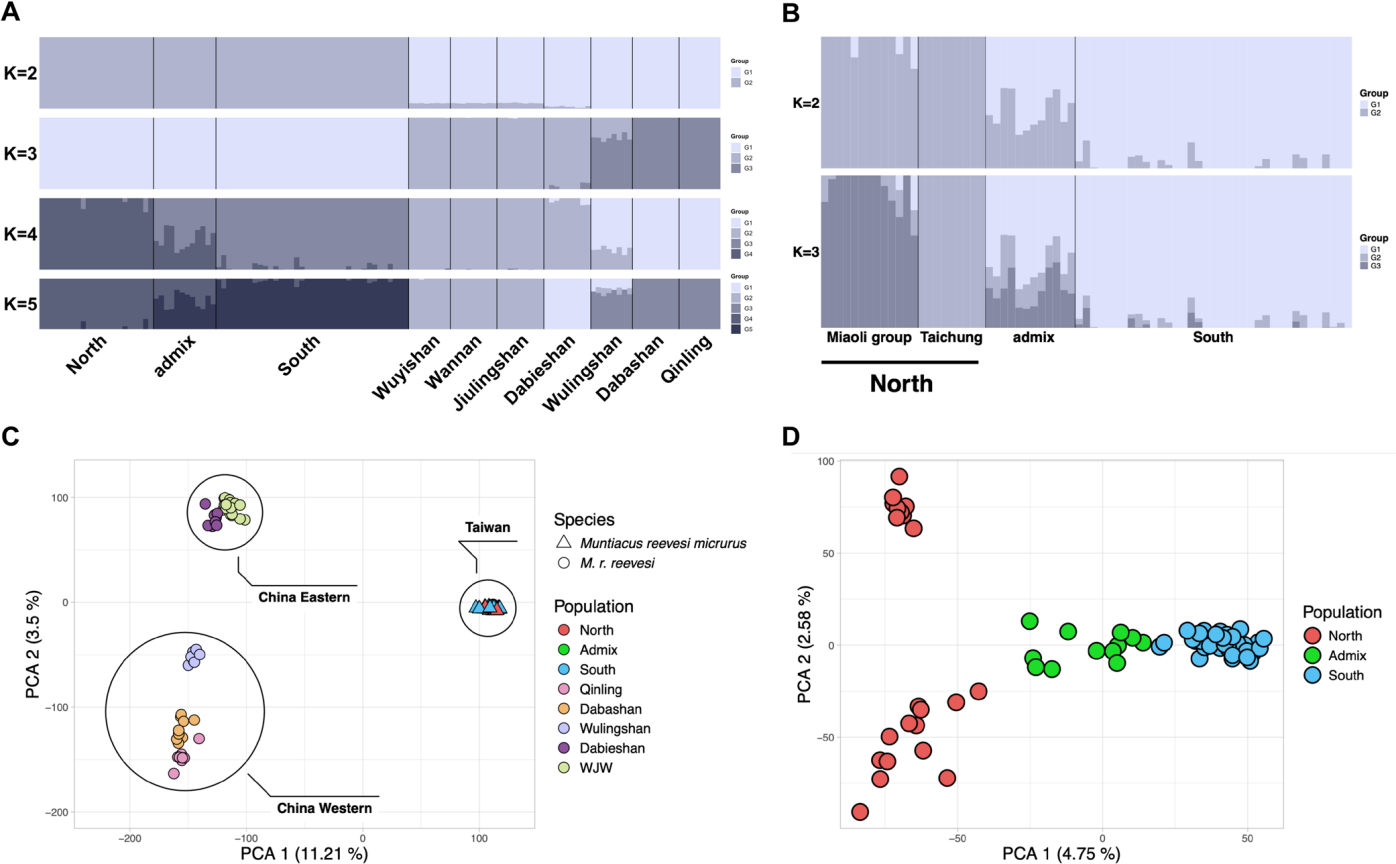
Population structure of Reeves's muntjac inferred from ADMIXTURE and PCA analyses. (A) ADMIXTURE analysis (*K* = 2–5) based on SNP data from Taiwanese and Chinese individuals. *K* = 2 provides the best‐supported clustering solution, separating Taiwanese and Chinese individuals; Chinese populations are further grouped into eastern (WJW: Wuyishan, Wannan, Jiulingshan, and Dabieshan) and western (Qinling, Dabashan, and Wulingshan) clusters, following Chen et al. (2024). (B) ADMIXTURE results for Taiwanese population (*K* = 2–3), with *K* = 2 representing the best‐supported model, reveal a north–south division and admixture in central populations. (C) Principal Component Analysis (PCA) showing genetic differentiation between Taiwanese and Chinese populations. (D) PCA showing genetic differentiation with Taiwanese populations. PC1 (4.75%) separates populations along a north–south geographic gradient, while PC2 (2.58%) further distinguishes two subgroups within the northern population.

As expected, both admixture and principal component analysis (PCA) revealed that Reeves's muntjac can be divided into two major genetic lineages, Taiwan and China (Figure 2). Consistent with this pattern, cross‐validation analysis suggested that *K* = 2 best explained the primary genetic structure (Figure S2), while higher *K* values revealed additional hierarchical subdivision. When *K* = 3, the muntjacs in China are further divided into eastern and western groups. As *K* increased to 4, the muntjacs collected from northern and southern Taiwan formed two distinct genetic clusters, separated by the Wu (Dadu) River and Zhoushui River, with admixed individuals observed in the intermediate region (Figure 1).

Phylogenetic analysis based on nuclear SNPs revealed patterns consistent with the Admixture results (Figure S3A). In contrast, the mitochondrial cytochrome *b* phylogeny showed that individuals from the same sampling locations often fell into different clades, indicating weak geographic structuring of mtDNA lineages (Figure S3B).

The *F*_ST_ based on nuclear SNPs between Taiwan and China ranges from 0.069 to 0.227 (Table 1). The Taiwanese Reeves's muntjac is genetically closest to the easternmost population of China's muntjac. Within Taiwan, where samples were collected from locations less than 300 km apart, the *F*_ST_ between the northern and southern populations is 0.036. This level of differentiation is comparable to that observed among muntjac populations in China, where *F*_ST_ values range from 0.017 to 0.056 with an average of 0.035, despite these samples being collected from locations over 1000 km apart. Interestingly, the *F*_ST_ derived from mtDNA shows a similar pattern as those from nuclear SNPs (Table 1B), indicating that the mitochondrial genome also retains signals of differentiation.

**TABLE 1:** Pairwise *F*_ST_ among Taiwanese and Chinese populations of Reeves's muntjac. (A) Nuclear loci (B) mitochondrial cytochrome b.

*F*_ST_		Taiwan			China			
		North	Admix	South	WJW	Dabieshan	Wulingshan	Dabashan
(A)								
Taiwan	Admix	0.025						
	South	0.036	0.016					
China	WJW	0.087	0.069	0.099				
	Dabieshan	0.174	0.140	0.198	0.017			
	Wulingshan	0.186	0.149	0.212	0.025	0.038		
	Dabashan	0.185	0.152	0.209	0.031	0.046	0.029	
	Qinling	0.204	0.169	0.227	0.036	0.056	0.039	0.029
(B)								
Taiwan	Admix	0.23						
	South	0.03	0.11					
China	WJW	0.11	0.10	0.07				
	Dabieshan	0.62	0.50	0.52	0.30			
	Wulingshan	0.41	0.24	0.33	0.15	0.51		
	Dabashan	0.41	0.25	0.33	0.16	0.45	0.18	
	Qinling	0.45	0.29	0.37	0.21	0.56	0.20	0.09

The estimated divergence time between China and Taiwan was 0.24 million years ago (MYA) with a 95% confidence interval (CI) of 0.08–0.54 MYA. Within Taiwan, the divergence between northern and southern populations occurred 25,000 years ago (95% CI: 8200–56,000) (Figure 1C).

### 3.2 Demography and Divergent History

**FIGURE 3:**
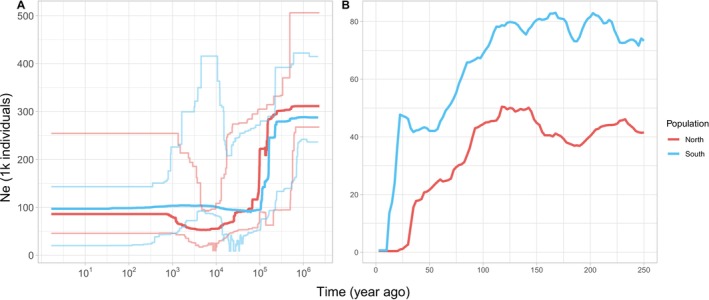
Demographic history of Taiwanese Reeves's muntjacs. Demographic changes in the northern (pink) and southern (blue) populations. (A) Stairway Plot inference of long‐term population size changes, showing a sharp decline ~200,000 years ago. Solid lines: Median estimates; dashed lines: 95% confidence intervals. (B) GONE analysis of recent history (~250 years), revealing a pronounced population decline ~125 years ago in both populations.

According to the stairway plot analysis, both northern and southern Taiwanese Reeves's muntjac populations experienced a population reduction beginning around 0.25 MYA (Figure 3A), which is consistent with their divergence from China's muntjac. Subsequently, population sizes remained relatively stable until a second decline occurred approximately 100–150 years ago, likely associated with recent human population expansion and increased anthropogenic pressures in Taiwan (Figure 3B).

**FIGURE 4:**
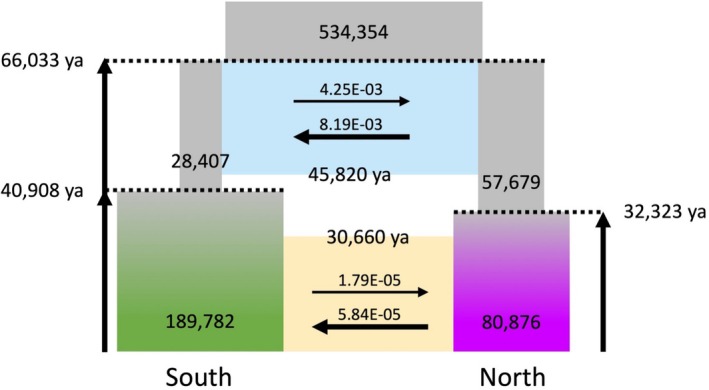
Demographic scenario of Taiwanese Reeves's muntjac inferred from fastsimcoal2 simulations. The best‐fit model supports a divergence between the northern and southern populations approximately 66,033 years ago, during Marine Isotope Stage 4 (MIS 4; ~71–57 ka), a period characterized by cold and arid climatic conditions in East Asia. These conditions may have facilitated southward dispersal and geographic isolation of the southern lineage. Substantial gene flow was detected after divergence, with asymmetric migration rates, higher from north to south (8.19 × 10^−3^) than from south to north (4.25 × 10^−3^). Gene flow ceased between 45,820 and 30,660 years ago, corresponding to MIS 3 (~43.5–34 ka), a relatively warm and stable climatic period. This warming may have allowed northward migration of the northern population, leading to the cessation of gene flow. Population expansion also likely occurred during this stable climatic interval. As MIS 2 (~29 ka) began, a cooling trend may have driven renewed southward migration from the northern population, reestablishing gene flow between the two lineages.

To further investigate the history of population differentiation in Taiwanese Reeves's muntjac, we applied a coalescent‐based simulation program fastsimcoal2 to estimate demographic parameters under 12 evolutionary scenarios (Figure S1, Table S6). The most supported demographic scenario incorporates population size changes and interrupted gene flow, which accounts for periods of both early and recent genetic exchange between the northern and southern populations (Figure 4). According to this model, the ancestor population of Taiwanese Reeves's muntjac was approximately 534,000 and the population divergence occurred 66,000 years ago. This divergence time, which accounts for post‐divergence migration, contrasts with the more recent split of ~25,000 years ago estimated via Bayesian inference (see Section 4 for a detailed comparison of these modeling approaches).

After divergence, the effective population size of the southern population declined to 28,407, while that of the northern population was 57,679. Subsequently, the southern population expanded to 190,000 around 41,000 years ago, while the northern population grew to 81,000 around 32,000 years ago. The model also indicates that there was gene flow between populations after divergence. Gene flow ceased from approximately 45,860 to 31,660 years ago. Afterwards, gene flow was reestablished. However, both instances of gene flow were asymmetric, with a higher migration rate from the northern population to the southern populations.

### 3.3 Factors That Cause Population Differentiation

**FIGURE 5:**
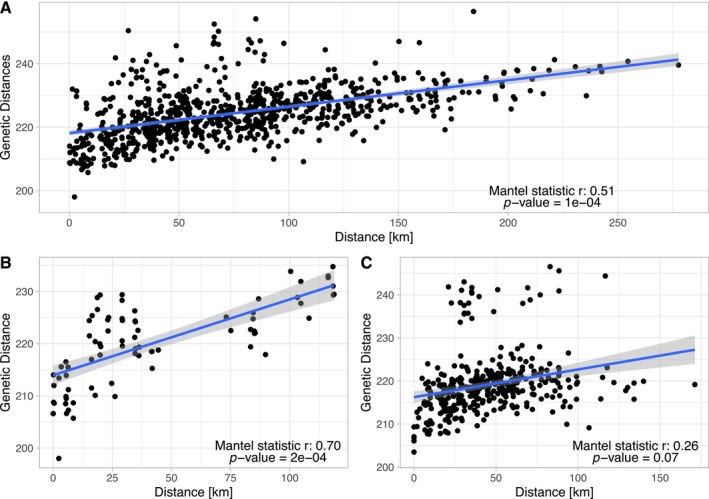
Regional patterns of isolation by distance in Taiwanese Reeves's muntjac. Mantel tests reveal a moderate correlation between genetic and geographic distances across all samples (A), a strong correlation in the northern population (B), and a weak, non‐significant correlation in the south (C), indicating spatial variation in isolation‐by‐distance dynamics.

Mantel test was used to assess the correlation between genetic and geographic distance, as well as the relationship between genetic distance and environmental factors among individuals. When all samples were analyzed together, a significant correlation between genetic and geographic distances was observed (*r* = 0.51, *p* < 10^−4^; Figure 5A). However, when analyzed separately, the northern population showed a strong correlation (*r* = 0.7, *p* < 10^−4^; Figure 5B), while no such correlation was found in the southern population.

Since the sampling of Taiwanese muntjac in this study spanned approximately 25 years, Mantel and partial Mantel tests were conducted to assess whether sampling time influenced genetic distance. The results indicated no significant relationship between genetic distance and sampling time (*r* = 0.01, *p* = 0.42).

Mantel test was also used to assess the correlation between genetic distance and environmental factors. The results showed that eight climatic variables, Bio2, Bio3, Bio4, Bio7, Bio14, Bio15, Bio17, and Bio19, were significantly correlated with genetic distance of Taiwanese muntjac populations (Table S7A). However, when geographic distance or least‐cost path was accounted for, the effects of these environmental variables were no longer significant. This suggests substantial overlap between environmental variables and geographic or least‐cost path distances. When the northern and southern populations were analyzed separately, the northern population showed a significant correlation between genetic distance and Bio3, Bio4, Bio14, Bio17, and Bio19, but these correlations were no longer significant after accounting for geographic distance or least‐cost path (Table S7B). In contrast, the southern population showed significant correlations with mean diurnal range (Bio2), temperature seasonality (Bio4), temperature annual range (Bio7), and precipitation of driest month (Bio14), and these associations remained significant even after controlling for geographic distance or least‐cost path (Table S7C).

**FIGURE 6:**
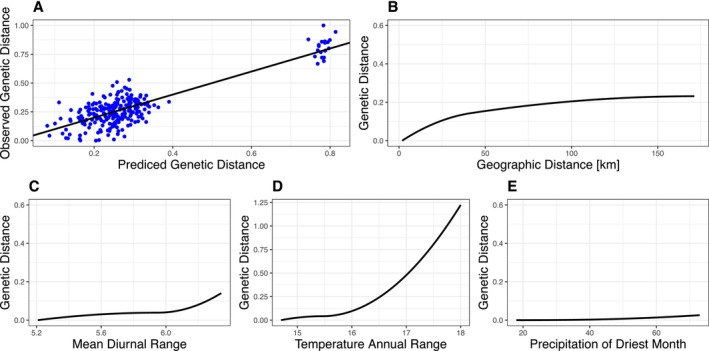
Generalized Dissimilarity Modeling (GDM) of genetic differentiation in the southern population of Taiwanese Reeves's muntjac. (A) Observed versus predicted genetic distances showing model fit. (B–E) Relative contributions of predictor variables: (B) Geographic distance, (C) Mean diurnal range (Bio2), (D) Temperature annual range (Bio7), and (E) Precipitation of the driest month (Bio14).

Generalized Dissimilarity Modeling (GDM) was used to evaluate the relative contribution of different environmental and geographic factors on genetic divergence. When the analysis included both northern and southern populations, the best model is least‐cost distance which explains 44.75% of genetic differentiation (Table S9A, Figure S4). For the northern populations, the best model considers only geographic distance which explains 59.84% of genetic differentiation (Table S9B, Figure S5). For the southern populations, the best model contains only temperature annual range (Bio7) which explains 62% of genetic variation (Table S9C, Figure 6).

**FIGURE 7:**
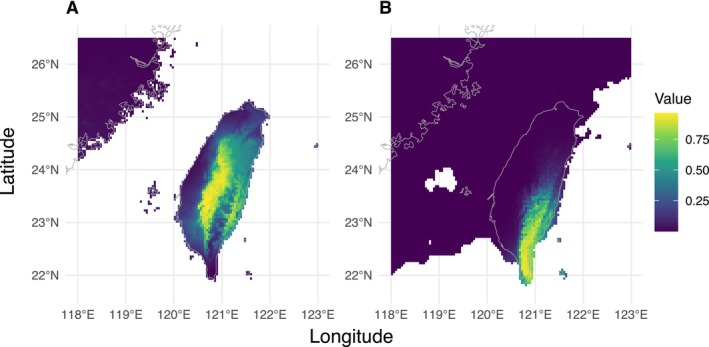
Predicted distribution of Taiwanese Reeves's muntjac from MAXENT modeling. (A) Current predicted distribution under contemporary climate. (B) Potential distribution during the Last Glacial Maximum (LGM). Models are based on species occurrence records and four bioclimatic variables: Bio2 (mean diurnal range), Bio7 (temperature annual range), and Bio15 (precipitation seasonality).

To model the distribution of the Taiwanese Reeves's muntjac in Maxent, we first removed highly correlated variables (pairwise correlations > |0.70|; Table S8), retaining only those most associated with genetic distance (Table S10). The final model included three predictors, mean diurnal range (Bio2), temperature annual range (Bio7), and precipitation seasonality (Bio15), which contributed 2.3%, 81.4%, and 16.2%, respectively. The model showed strong predictive accuracy (AUC = 0.89; Figure S7). The niche of the Taiwanese Reeves's muntjac during the Last Glacial Maximum (LGM) appears to have contracted toward the southeastern region of the island, particularly around the southern tip (Figure 7).

To assess niche differentiation between Taiwanese muntjac populations, we conducted PCA‐env using three uncorrelated bioclimatic variables (Bio2, Bio7, Bio15). The first two axes explained 96.15% of climatic variation (PC1 = 57.63%, PC2 = 38.52%) (Figure S6A). Southern individuals occupied a narrower climatic space, while northern individuals spanned broader conditions. The niche equivalency test (*D* = 0.13, *p* = 0.05; Figure S6B) indicated statistically distinct niches, but the niche similarity test was not significant (*p* = 0.86; Figure S6C), suggesting the difference may not exceed background environmental variation. These results imply that divergence is more likely shaped by geographic separation and environmental gradients than by adaptive niche differentiation.

### 3.4 Putative Regions Under Selection

Using PCadapt and BayeScan, 90 and 14 SNPs potentially under positive selection were identified, respectively, with 10 SNPs detected by both methods (Table S11). Within a 150 kb region surrounding these SNPs, 154 protein‐coding genes were identified. Gene enrichment analysis revealed a significant Gene Ontology (GO) term, GO:0004623, which is associated with phospholipase A2 (PLA2) activity (Table S12). PLA2 is involved in various biological functions, including signal transduction, lipid mediator production, and membrane remodeling, and plays a critical role in responding to environmental stressors such as temperature fluctuations and oxidative stress (Murakami et al. 2017). Among these, responding to temperature fluctuations is the most interesting, as the most prominent environmental factor associated with Taiwanese muntjac divergence is temperature annual range (Bio7), which is the indicator of seasonal temperature variation.

The mouse orthologs of these genes are Pafah1b3, Pla2g4d, Pla2g4e, and Pla2g4f. According to phenotype enrichment analysis (modPhEA) (Weng and Liao 2017), these genes were involved in ion homeostasis (Pla2g4f) and physiological strength and integument phenotype (Pla2g4e) (Table S13).

## 4 Discussion

We sequenced 71 individuals of Taiwanese Reeves's muntjac ( *Muntiacus reevesi micrurus* ) using ddRAD‐seq and incorporated 60 whole‐genome sequences of *M. reevesi* from China to facilitate comparative analyses. Our genomic analyses reveal a clear and unexpected genetic subdivision within the Taiwanese Reeves's muntjac, a finding that challenges previous mitochondrial studies suggesting a lack of population structure. By leveraging high‐resolution SNP data alongside mainland Chinese populations, we identified a deep divergence dating back to the late Pleistocene, followed by a subsequent north–south split within Taiwan approximately 66,000 years ago. This structure is not merely a product of historical isolation but is significantly shaped by contemporary environmental heterogeneity, specifically temperature annual range (Bio7), and asymmetric gene flow. Our results demonstrate that even in highly mobile generalist herbivores, climatic gradients can drive local adaptation and restrict movement, providing a more nuanced understanding of how landscape genetics and evolutionary ecology intersect in montane island ecosystems.

### 4.1 Divergent History of Muntjac

The divergent history of Reeves's muntjac in East Asia coincides with past climatic fluctuations and geological events. Bayesian inference indicates that the divergence between Taiwanese and Chinese muntjac populations occurred 0.24–0.25 MYA, at the onset of Marine Isotope Stage 7 (MIS 7; ~0.24–0.19 MYA), likely driven by sea‐level rise during this warm interglacial period (Sato et al. 2017). This timing is consistent with demographic analyses showing a sharp population decline in Taiwanese muntjac between 0.3 and 0.25 million years ago, likely due to a founder effect associated with the colonizing lineage. Furthermore, the nucleotide diversity of Taiwanese muntjac is only half that of their Chinese counterparts, supporting a scenario of reduced genetic diversity following colonization.

The divergence between the northern and southern Taiwanese muntjac populations was estimated at approximately 66,000 years ago, during MIS 4 (∼71–57 ka), a period characterized by cold and dry climatic conditions in East Asia (Doughty et al. 2021). This initial split was followed by gene flow between the two populations. We observed that this divergence estimate contrasts with that inferred from Bayesian inference (Figure 1C), which does not account for post‐divergence migration and instead estimates a more recent split at ~25,000 years ago. Interestingly, when migration was not allowed in fastsimcoal2 simulations, the inferred divergence also shifted to ~20,000 years ago (Table S6), similar to the Bayesian result. These contrasts highlight that incorporating gene flow yields a more realistic reconstruction of the muntjac's demographic history, capturing both the divergence and subsequent genetic exchange between northern and southern populations.

This estimated timeframe also coincides with the major tectonic uplift and surface emergence of southern Pingtung, including the Hengchun Peninsula during the Late Pleistocene (129,000–11,700 years ago) (Giletycz et al. 2019). Species distribution modeling based on LGM climatic conditions suggests that suitable habitats for the Taiwanese muntjac were primarily concentrated in the southern tip of the island (Figure 7). Therefore, the newly emerged Hengchun Peninsula may serve as a refugium for the southern population and contribute to its isolation from the northern lineage. Substantial gene flow was detected after population divergence. The migration rate from north to south was two‐fold higher than vice versa.

Gene flow ceased between 45,820 and 30,660 years ago, corresponding to the mid‐stage of MIS 3 (43.5–34 ka), a period characterized by relatively stable and warm climatic conditions (Wang et al. 2021; Wei et al. 2025). The warmer climate may have facilitated the northward migration of the northern population, resulting in the interruption of gene flow. Additionally, this climatic stability may have contributed to population expansion during this interval. Toward the end of MIS 3 and beginning of MIS 2 (~2.9 ka), a gradual cooling trend began, which may have driven southward migration of the northern population and subsequently reestablished gene flow between populations.

### 4.2 Factors That Drive Muntjac Differentiation in Taiwan

Although geographic distance is the primary factor explaining genetic differentiation when all Taiwanese muntjac samples are considered, our results suggest that climatic factors may also contribute to the observed population structure, particularly between the north and south. Notably, despite the sampling sites in Taiwan being less than 300 km apart, the observed *F*_ST_ (0.036) between northern and southern populations is comparable to the differentiation among Chinese populations (*F*_ST_ = 0.017–0.056, mean = 0.035), where samples span more than 1000 km. While this elevated differentiation over a short distance could partially reflect a stronger effect of IBD due to Taiwan's rugged montane topography compared to the mainland, our results suggest that additional environmental factors may also contribute to population differentiation.

Significant correlations were found between genetic distance and several climatic variables, including mean diurnal range (Bio2), temperature seasonality (Bio4), temperature annual range (Bio7), and precipitation of the driest month (Bio14), in the southern population, even after controlling for geographic distance. GDM modeling identified temperature annual range (Bio7) as the strongest predictor of genetic variation in the south, explaining 62% of the observed divergence. Furthermore, while niche similarity tests showed no evidence of large‐scale adaptive niche divergence, a significant niche equivalency test confirmed that these populations occupy environmentally distinct regions, suggesting that climatic heterogeneity is an additional driver of the observed structure. Together, these results support the hypothesis that both geography and environmental heterogeneity shape the north–south differentiation of Taiwanese Reeves's muntjac, likely via local adaptation or climate‐driven restrictions on gene flow. The observed asymmetrical gene flow potentially aligns with these regional differences in temperature. The identification of selection in thermal‐adaptation genes (PLA2) suggests that local adaptation may occur at the genomic level, even if broad ecological niche shifts are not yet detectable. Under this hypothesis, southern individuals might experience subtle physiological constraints when moving into cooler northern habitats, whereas northern individuals may possess broader tolerances for warmer conditions. This mechanism could contribute to the net southward gene flow; however, further physiological studies are needed to confirm whether these climatic gradients directly impose filters on migration or if other demographic factors are primarily responsible for the observed asymmetry.

### 4.3 Potential Thermogenic Adaptation Through PLA2‐Mediated Pathways

Interestingly, we identified a group of genes under putative positive selection that are significantly enriched for phospholipase A2 (PLA2) activity (GO:0004623), suggesting an adaptive role in environmental responsiveness. PLA2 plays a critical role in responding to environmental stressors such as temperature fluctuations and oxidative stress (Murakami et al. 2017). For example, although their functional roles remain unclear, variants of *PLA2G2A*, a member of the PLA2 protein family, identified in indigenous human populations from Siberia show signs of positive selection and are rare outside this region, suggesting region‐specific selective pressures related to cold adaptation in Indigenous Siberian groups (Hallmark et al. 2018).

Among the candidate PLA2 genes, *PLA2G4D* and *PLA2G4E* are cytosolic PLA2 (cPLA2), which hydrolyze membrane phospholipids, releasing free fatty acids, particularly arachidonic acid (AA) (Leslie 2015), which is then metabolized by cyclooxygenases (COX‐1 and COX‐2) to produce prostaglandin H2 (PGH2), the central precursor of various bioactive prostaglandins, including PGE2 (Vasilakaki et al. 2016). While PGE2 is best known for inducing fever in response to pathogen‐triggered inflammation (Lazarus et al. 2007), emerging evidence suggests it also contributes to thermoregulation under cold stress (Foster et al. 2015). In healthy individuals exposed to environments below the thermoneutral zone, PGE2 appears to facilitate heat production by disinhibiting thermogenic pathways involving brown adipose tissue activation, shivering, and vasoconstriction (Foster et al. 2015). These findings imply that the cPLA2‐PGE2 pathway may serve as a key mechanism for coping with both biotic and abiotic thermal challenges.

In addition to those cPLA2, the third PLA2 candidate *PLA2G4F* is a mitochondrial PLA2. During cold exposure, mPLA2 can release long‐chain fatty acids from the inner mitochondrial membrane. These fatty acids compete with purine nucleotides for binding to uncoupling protein 1 (UCP1), facilitating proton re‐entry into the mitochondrial matrix and promoting thermogenesis (Roesler and Kazak 2020). Therefore, *PLA2G4F* may be involved in stimuli that drive catabolic processes and the uncoupling of oxidative phosphorylation, an efficient thermogenic mechanism during cold exposure.

### 4.4 Parallel North–South Divergence Across Mammals in Taiwan

Based on nuclear SNPs, we observed clear genetic differentiation among the northern, southern, and admixed populations (Table 1A). In contrast, previous analyses using mitochondrial cytochrome b sequences did not detect strong population structure. However, when mtDNA sequences are re‐grouped according to the population boundaries defined by nuclear genomic data, patterns of mitochondrial differentiation become evident (Table 1B). For instance, the *F*_ST_ between the northern and southern populations based on mtDNA is 0.030, similar in magnitude to that derived from nuclear markers (*F*_ST_ = 0.036). This suggests that mitochondrial data, although more limited in resolution, still capture meaningful differentiation when interpreted within the correct population framework.

More interestingly, the north–south split in Reeves's muntjac is strikingly similar to that observed in several other endemic mammals, including the mole‐shrew, Formosan wood mouse, Formosan serow, and sambar deer (Hsu et al. 2001; Horng et al. 2003; Yuan et al. 2006; Li et al. 2023). Despite differences in ecological niches and dispersal abilities, these species share congruent phylogeographic boundaries, with divergence often occurring near the headwaters of the Zhoushui River, in the vicinity of Hehuanshan in Taiwan's central mountain region. This consistency across taxa suggests that their population structures may have been shaped not only by shared historical processes, such as isolation in Pleistocene glacial or interglacial refugia, but also by common geographic and environmental constraints. The repeated emergence of a north–south split points to a consistent pattern in which climatic gradients and topographic barriers have played a central role in driving intraspecific divergence across Taiwan's montane ecosystems. While previous studies have emphasized the role of historical or geographic isolation (Oshida et al. 2006; Yuan et al. 2006), our results highlight the additional role of environmental heterogeneity, particularly climatic variation, in shaping the divergence of Taiwanese muntjac.

Although ddRAD‐seq is a cost‐effective way to genotype many individuals at high depth, it targets only a small, non‐random fraction of the genome near restriction sites (Martchenko and Shafer 2023). As a result, loci are unevenly distributed and may miss key functional or regulatory variants, particularly when adaptation is polygenic. Therefore, while our outlier scans highlight plausible candidates (e.g., PLA2‐associated genes), additional adaptive signals may remain undetected due to the limited genomic coverage of ddRAD‐seq. Whole genome sequencing, ideally with broad geographic sampling, would provide more complete and uniform variant discovery, improve resolution for selection mapping, and better characterize the genomic architecture of climate‐associated adaptation in Taiwanese muntjac and other mammals.

## 5 Conclusion

Using genome‐wide ddRAD‐seq SNPs from 71 Taiwanese Reeves's muntjac and comparative Chinese genomic data, we reveal that Taiwanese muntjac are deeply diverged from mainland populations and further subdivided into clear north–south genetic clusters within Taiwan. This subdivision aligns broadly with the Wu (Dadu) and Zhoushui River boundary and is shaped by both geography and climate, with temperature annual range (Bio7) emerging as a key environmental correlate of differentiation. Demographic analyses further suggest asymmetric gene flow predominantly from north to south, consistent with environmentally mediated barriers to dispersal across Taiwan's climatic gradients. Finally, selection scans identify PLA2‐associated genes as candidate targets potentially linked to thermal responsiveness, offering a plausible genomic foothold for climate‐associated local adaptation. Together, our results show that environmental heterogeneity and topography jointly contribute to muntjac divergence and support the broader view that shared climatic gradients can drive parallel north–south structuring in Taiwan's montane mammals.

## Author Contributions

**Yi‐Lun Peng:** formal analysis (lead), methodology (equal), visualization (lead), writing – original draft (equal). **Hsuan‐Wien Chen:** resources (equal), writing – review and editing (supporting). **Shih‐Wei Chang:** resources (equal), writing – review and editing (supporting). **Chun‐Yi Hsiao:** methodology (equal). **Hurng‐Yi Wang:** conceptualization (lead), resources (lead), supervision (lead), writing – original draft (equal).

## Funding

This study was supported by the National Science and Technology Council (113‐2327‐B‐002‐003, 114‐2327‐B‐002‐005, and 114‐2621‐B‐002‐004‐).

## Conflicts of Interest

The authors declare no conflicts of interest.

## Supporting information


**Data S1:** ece373283‐sup‐0001‐Figure.docx.

**Data S2:** ece373283‐sup‐0002‐Table.xlsx.

## Data Availability

The datasets generated and analyzed during the current study are available in the NCBI Sequence Read Archive (SRA) repository under the accession number PRJNA1276262, and are included in this published article and its Supporting Information.
