# Supplementary material for: Beyond Geography: Climatic Gradients Shape Reeves's Muntjac Population Structure in Taiwan
Source: Ecol Evol. 2026 Mar 18;16(3):e73283. doi: 10.1002/ece3.73283 (PMC13093641; doi:10.1002/ece3.73283)
Supplement: Supplementary file 1 — Data S1: ece373283‐sup‐0001‐Figure.docx. [file ECE3-16-e73283-s001.docx]

**Figure S1. Demographic scenarios modeled using fastsimcoal2.
Schematic overview of the 12 demographic models simulated in fastsimcoal2. T1 represents the divergence time, while T2 and T3 indicate the timing of population size changes.**


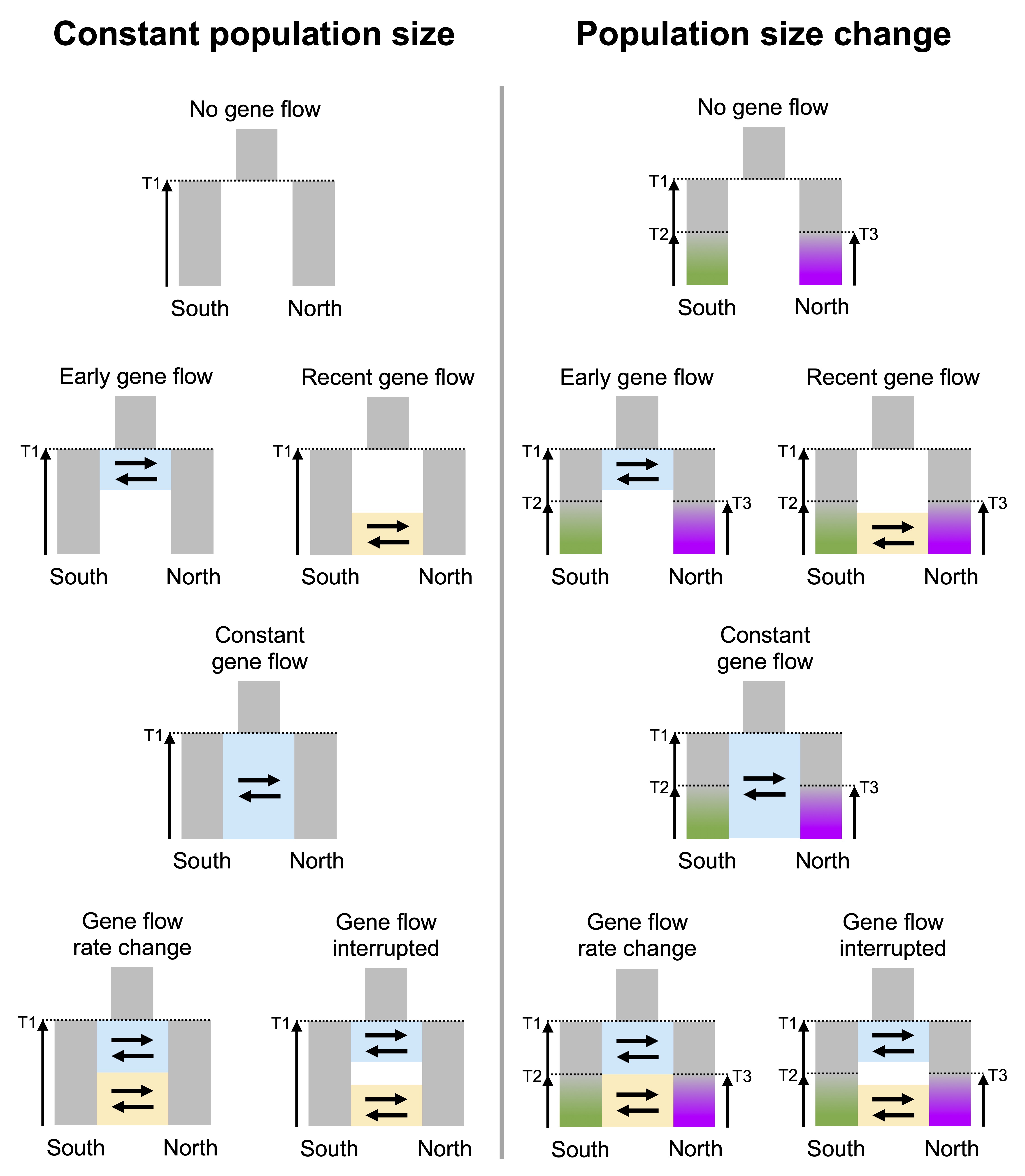

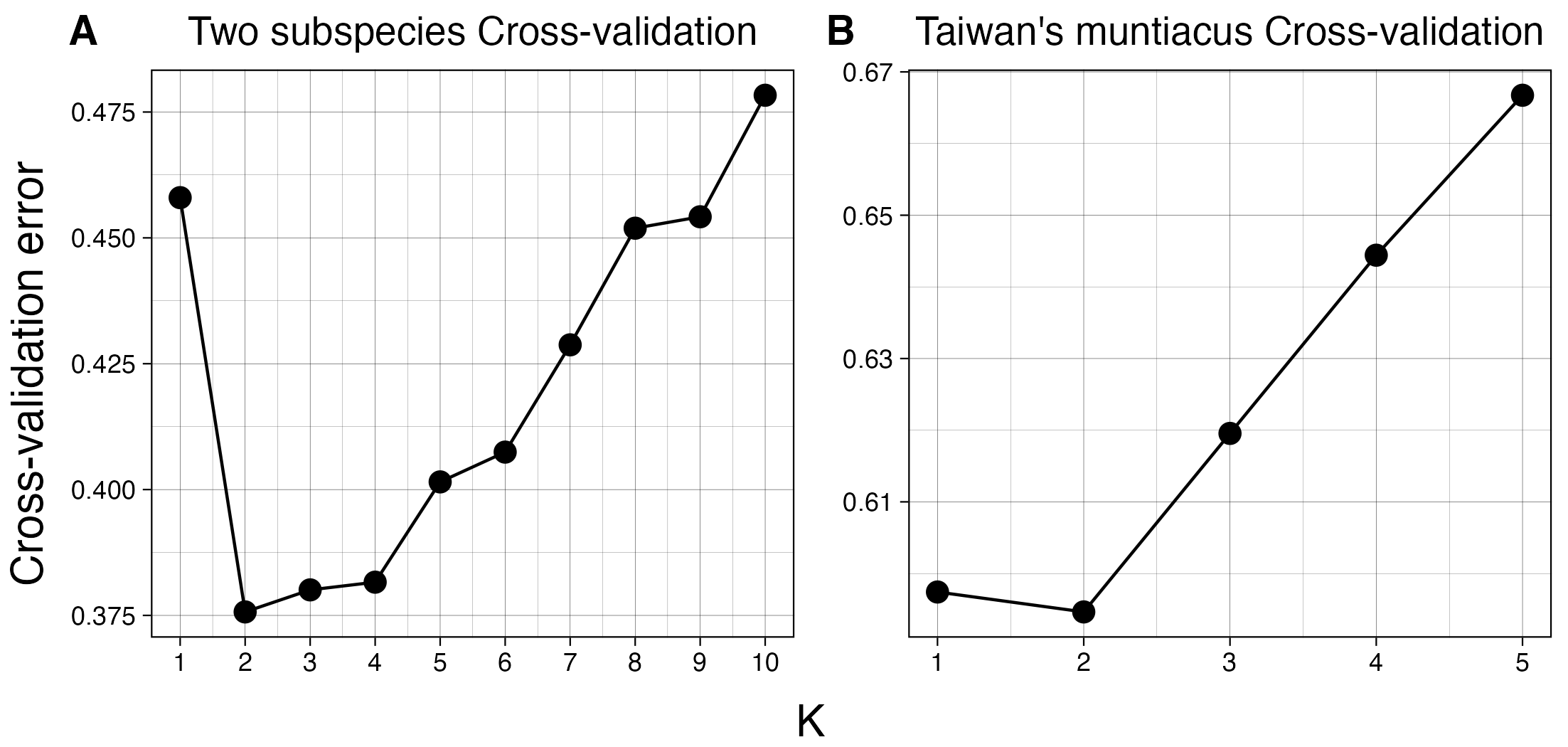


**Figure S2. ADMIXTURE cross-validation results.**

(A) Cross-validation error for the two subspecies dataset, with the lowest value at K = 2.

(B) Cross-validation error for the Taiwanese muntjac dataset, also indicating an optimal K = 2.

**Figure S3. Phylogenetic relationships among Taiwanese and Chinese Reeves’s muntjac.
(A) Maximum likelihood tree constructed using nuclear SNP data.
(B) Neighbor-joining tree based on mitochondrial cytochrome b sequences using the Kimura 2-parameter model.
Taiwanese samples are color-coded by genetic cluster: northern (pink), admixed (green), and southern (blue). Chinese samples are grouped into eastern (gray) and western (brown) populations.**

**Figure S4. Generalized Dissimilarity Modeling (GDM) results for genetic differentiation in Taiwanese Reeves’s muntjac.
(A) Observed vs. predicted genetic distances, indicating model fit.
(B–E) Relative contributions of predictor variables: (B) Least-cost path distance, (C) Temperature annual range (Bio7), (D) Precipitation seasonality (Bio15), and (E) Mean diurnal temperature range (Bio2).**

**Figure S5. Generalized Dissimilarity Modeling (GDM) results for genetic differentiation within the northern population of Taiwanese Reeves’s muntjac.
(A) Observed vs. predicted genetic distances, indicating model fit.
(B–E) Relative contributions of predictor variables: (B) Geographic distance, (C) Mean diurnal temperature range (Bio2), (D) Temperature annual range (Bio7), and (E) Precipitation of the driest month (Bio14).**

**Figure S6. PCA-env analysis of ecological niche differentiation between northern and southern muntjac populations.
(A) Climatic niche distribution based on PCA-env using Bio2, Bio7, and Bio15. Solid lines indicate 100% of the niche range; dashed lines show 50%. Blue shading indicates niche overlap.
(B) Niche equivalency test comparing observed and randomized niche overlap (Schoener’s D).
(C) Niche similarity test showing observed D (red line) against a null distribution.**

**Fig. S7. ROC curve of the MAXENT model.**

The x-axis represents 1 - specificity (Fractional Predicted Area), and the y-axis represents sensitivity (1 - Omission Rate). The blue line indicates the curve for the training data, with a corresponding AUC (Area Under the Curve) of 0.891. The red line represents the baseline for random prediction (AUC = 0.5).
